# Impact of clerkship in the attitudes toward psychiatry among Portuguese medical students

**DOI:** 10.1186/1472-6920-10-56

**Published:** 2010-08-02

**Authors:** Miguel Xavier, José C Almeida

**Affiliations:** 1 Department of Mental Health, CEDOC, Faculdade de Ciências Médicas, Universidade Nova de Lisboa, Portugal

## Abstract

**Background:**

Given the shortage of human resources and the launching of a new Mental Health Plan, recruitment of psychiatrists is currently a major concern in Portugal, as well as in several other countries. Medical students' attitude toward psychiatry has been pointed as a predictor of recruitment. This study aims to evaluate the medical students' perception of psychiatry before and after a clerkship, and the impact on their intention to pursue psychiatry as a future specialty option.

**Methods:**

Two self-report questionnaires were administered to all 6^th ^year students in a medical school in Lisbon, before and after a 4-weeks full-time psychiatric clerkship, in order to evaluate attitudes toward psychiatry and intention to follow psychiatry in the future. Statistical analysis included Wilcoxon and Chi-square tests.

**Results:**

153 students (60.8% female) filled in both questionnaires (no dropouts). After the clerkship, there was a significant improvement regarding the overall merits of psychiatry, efficacy, role definition and functioning of psychiatrists, use of legal powers to hospitalize patients and specific medical school factors. There was also a significant increase of students decided or considering the possibility to take a residency in psychiatry.

However, perceptions of low prestige and negative pressure from family and peers regarding a future choice of psychiatry remained unchanged in about one-third of the students.

**Conclusions:**

The results indicate clearly that the clerkship had a favorable overall impact on the student attitude towards psychiatry, as well as in the number of students considering a future career in psychiatry. Attitudes toward psychiatry seems a promising outcome indicator of the clerkship's quality, but further research is needed in order to assess its reliability as a sound predictor of recruitment.

## Background

Portugal, like many other countries [1-5], is currently struggling with a shortage of psychiatrists in its public health system. Though not with the severity found in other countries, the ratio of psychiatrists per 100,000 inhabitants found in Portugal, a country with 10 million inhabitants, is still clearly inferior to that existing in the more developed countries in the European Union.

According to the available data [6], Portugal's ratio (4,7/100.000) is in stark contrast with those found in Central and North European countries (Sweden - 20/100.000, France - 22/100.000, UK - 11/100.000), as well as with Southern European countries (Italy - 9,8/100.000, Greece: 15/100.000).

This issue, resulting from a combination of several factors, has recently worsened. Firstly, the small number of physicians graduating from Portuguese medical schools, a direct result of the Governmental policy of restricted admission (*numerus clausus*), which has been the norm over the past 30 years. The number of graduating physicians has dwindled so acutely that the yearly Internship allocations have seen an excess of vacancies. Secondly, due to the scarcity of physicians in a given field, particularly in primary care, the Government has allocated substantial amounts of vacancies to fields like family medicine (accounted for 30% of vacancies in the 2009), thus reducing the amount available for other fields.

Thirdly, the changes in the management model of the National Health Service, together with a booming private health sector and health insurance companies, have contributed to the existing flow of physicians from the National Health Service toward private enterprises, where the salary is higher, thus further reducing the number of physicians available in the National Health System.

Lastly, if the advanced average age of the psychiatrists is taken into consideration, it becomes clear that within the next 5-10 years their numbers will decline sharply, with added risk to the availability and delivery of mental healthcare in the public sector.

The scarcity of medical resources, worrying in itself, is becoming a challenging issue given the reform of the Portugal's Mental Healthcare System, under way since 2007. The 'National Mental Health Plan 2007-2016', developed by the Ministry of Health with input from the World Health Organization, aims at the development of mental healthcare services across the Country, in order to ensure a comprehensive coverage of the population, regardless of their location. The development of this network requires the creation of new services in regions with greater need, in order to guarantee the implementation of new healthcare programmes in fields such as severe mental illness, child psychiatry, liaison with primary care and psychogeriatrics. Though the 'National Mental Healthcare Plan 2007-2016' underlines the importance of a multidisciplinary approach and the importance of non-physicians in the local Mental Health teams, there is an undeniable shortage of psychiatrists and child psychiatrists in the public system, with particular severity outside the great urban areas, and a clear trend toward its growth in the mid-range.

In the present context of simultaneous dwindling of medical resources and increased need, it is crucial that greater efforts are made to improve the recruitment of new psychiatrists, which must begin with undergraduate medical training.

Portuguese medical students are not immune to the factors which, according to the literature, seem to push student away from pursuing a medical career in psychiatry, such as: low status when compared with other specialties; seemingly less scientific, stigma, lower consideration among peers, lower financial benefits, and 'bad-mouthing' by medical specialists from other fields [7-9].

The impact of such factors may only be reduced through processes that induce a change to the negative outlook toward psychiatry. According to the existing literature on the subject, the carrying out of clerkships can produce significant changes to the student's attitude toward psychiatry, which is why their impact is being studied in various socio-economic environments.

Several studies seem to underline the positive effect a clerkship has on the student's attitude towards psychiatry. This effect is particularly visible in clerkships that allow students to participate in the direct provision of healthcare, to witness patient recovery, and to join the staff group [10-15]. In Portugal, these clerkships, which take place in the final year of undergraduate medical training, have suffered profound changes and standardization by Governmental initiative. This initiative was supported by the creation of guidelines[16] based on a set of well known international documents on medical education[17-19]. However, little is known on the real impact of these curriculum changes on the learning process, on performance evaluation, or the student's attitude towards the various fields of clinical medicine. This study seeks to assess the medical students' perception of psychiatry and ascertain the impact of this new model of clerkship in this perception, with particular emphasis on the perspective of psychiatry as a future specialty option.

## Methods

The population of this longitudinal 'before and after' study was the student body of the Faculty of Medical Sciences of the New University of Lisbon attending their final year of Medical training, composed entirely of clerkships (Internal Medicine, Surgery, Paediatrics, Obstetrics, Public Health, Family Medicine and Mental Health).

The Mental Health clerkship has duration of 4 weeks, on a full-time basis, and includes a module of theoretical and practical seminars (25 h) and a module for clinical practice (75-100 h), in which students can rotate through hospitalization valences, outpatient clinics, day hospital, child psychiatry, liaison psychiatry, rehabilitation and psychiatric emergency. As a general principle, the clerkship was renewed in order to avoid an overemphasis on acute medicine and inpatient settings. This approach contrasts with the traditional inpatient setting where the teaching of psychiatry takes place during the fifth year of the course, with a particular emphasis on psychopathology and clinical diagnosis of major psychiatric disorders.

At the beginning of the psychiatry clerkship, the study was presented by one of the authors (MX) to all 6th year students, who were invited to participate voluntarily, with a guarantee of total anonymity.

Every student agreeing to participate in this study after informed consent was requested to answer a questionnaire on attitudes toward psychiatry, to be filled in on the first and last days of the clerkship. In order to avoid a potential positive bias caused by the students' fear that negative responses in the questionnaire could lead to less favourable treatment during the clerkship or the final grade, the completed pre-clerkship questionnaires remained in a closed envelope and in possession of a student representative for each class during the entire probation period, while the post-clerkship questionnaires were only opened after the final exam. Ethical approval was obtained from the Institutional Executive Board of the Faculty of Medical Sciences.

A self-report questionnaire designed by Balon was the principal tool utilized, which had already been used in several studies in countries with a different cultural context [5,20,21]; formal permission to use the questionnaire in this study was obtained after contact with the author. The questionnaire was translated into Portuguese from the original English version and back-translated by an English native fluent in Portuguese. The original version of the questionnaire was scrutinized in a discussion group including the principal investigator and the medical students, in order to adjust issues pertaining to the Portuguese culture and educational context.

The questionnaire contains 29 multiple choice questions (taking 10-15 minutes to complete), which assess medical students' attitude toward psychiatry in the following six domains: i. overall merits of psychiatry, ii. efficacy, iii. role definition and functioning of psychiatrists, iv. possible abuse and social criticism, v. career and personal reward and vi. specific medical school factors. Questions 25 and 26 were not assessed in this study, as there was no previous close contact with residents and tutors that could allow for a pre-clerkship assessment. In each question the student had to choose one of four choices according to their degree of agreement with the contents of the item: 'strongly agree', 'moderately agree', 'moderately disagree' and 'strongly disagree'.

Along with this questionnaire, medical students were also asked about how seriously they were considering psychiatry as a future career choice, in a short questionnaire with five items. Data has been described by means of percentages for each item in the questionnaire. In the analysis, the Balon's questionnaire was treated as an ordinal scale of measurement. A comparison between the results obtained before and after the clerkship was carried out with the Wilcoxon test (attitudes towards psychiatry) and the Chi-square test (intention to pursue psychiatry as a future option). The software package PASWStatistics 18 for MAC/OX was used for analysis.

## Results

All students invited agreed to participate in the study. 153 students (93 females, 60 males) filled in the questionnaires, both at starting and at the end of the clerkship. There were no dropouts between the assessments. The results of the items assessing students' attitudes toward psychiatry are presented in Table 1, Table 2, Table 3, Table 4, Table 5 and Table 6. Regarding the reporting of results, it was assumed that students agreed with a statement if they chose 'moderately agree' or 'strongly agree'.

**Table 1:** Overall merits of psychiatry (percentage of students)

	Before (%)				After (%)				Statistics	
**Question**	**SA**	**MA**	**MD**	**SD**	**SA**	**MA**	**MD**	**SD**	**Z**	**p**

1. Psychiatric research has made good strides in advancing care of the major mental disorders.	41.9	51.6	6.5	0.0	52.3	45.7	2.0	0.0	2.03	.04
2. Psychiatry is a rapidly expanding frontier of medicine.	13.7	58.8	26.2	1.3	20.3	66.6	12.4	0.7	2.62	.008
3. Psychiatry is unscientific and imprecise.	4.0	23.5	50.3	22.2	0.6	11.1	49.7	38.6	-3.57	.0003

SA = Strongly agree; MA = Moderately agree; MD = Moderately disagree; SD = Strongly disagree.

**Table 2:** Efficacy (percentage of students)

	Before (%)				After (%)				Statistics	
**Question**	**SA**	**MA**	**MD**	**SD**	**SA**	**MA**	**MD**	**SD**	**Z**	**p**

4. If someone in my family was very emotionally upset and the situation did not seem to be improving, I would recommend a psychiatric consultation.	85.6	11.1	1.3	2	87.6	11.7	0.7	0.0	1.0	.32,ns
5. Psychiatric consultation for medical or surgical patients is often helpful.	52.3	41.1	5.9	0.7	65.3	30.7	3.3	0.7	2.11	.03
6. Psychiatric treatment is helpful to most people who receive it.	31.3	57.5	10.5	0.7	51.6	43.8	4.6	0.0	3.58	.0003

SA = Strongly agree; MA = Moderately agree; MD = Moderately disagree; SD = Strongly disagree.

**Table 3:** Role definition and functioning of psychiatrists (percentage of students)

	Before (%)				After (%)				Statistics	
**Question**	**SA**	**MA**	**MD**	**SD**	**SA**	**MA**	**MD**	**SD**	**Z**	**p**

7. Psychiatry is not a genuine and valid branch of medicine.	1.3	1.3	15	82.4	5.9	1.3	5.2	87.6	0.4	.68, ns
8. Most psychiatrists are clear, logical thinkers.	18.3	53.6	24.8	3.3	24.2	61.4	11.8	2.6	2.32	.02
9. With few exceptions, clinical psychologists and social workers are just as qualified as psychiatrists to diagnose and treat emotionally disturbed persons.	0.7	8.5	41.1	49.7	1.3	3.9	26.2	68.6	2.67	.007
10. Among mental health professionals, psychiatrists have the most authority and influence.	41.2	40.5	12.4	5.9	47.7	39.2	11.8	1.3	1.66	.09,ns
11. Psychiatrists are too frequently apologetic when teaching psychiatry.	24.8	54.9	16.4	3.9	14.4	36.6	41.8	7.2	4.75	.0000
12. Psychiatry is too ''biologically'' minded and not attentive enough to the patient's personal life and psychological problems.	2	5.9	32	60.1	0.7	10.5	24.8	64	0.06	.95,ns
13. Psychiatry is too analytical, theoretical, and psychodynamic, and not attentive enough to patient's physiology.	1.3	8.5	51.6	38.6	0.0	2.6	41.8	55.6	-3.34	.0008

SA = Strongly agree; MA = Moderately agree; MD = Moderately disagree; SD = Strongly disagree.

**Table 4:** Possible abuse and social criticism (percentage of students)

	Before (%)				After (%)				Statistics	
**Question**	**SA**	**MA**	**MD**	**SD**	**SA**	**MA**	**MD**	**SD**	**Z**	**p**

14. Psychiatrists frequently abuse their legal power to hospitalize patients against their will.	5.9	2.6	30.1	61.4	2	0.7	25.5	71.9	2.26	.02
15. On average, psychiatrists make as much money as most other doctors.	19	47	27.5	6.5	19.6	53	24.8	2.6	1.36	.17,ns

SA = Strongly agree; MA = Moderately agree; MD = Moderately disagree; SD = Strongly disagree.

**Table 5:** Career and personal reward (percentage of students)

	Before (%)				After (%)				Statistics	
**Question**	**SA**	**MA**	**MD**	**SD**	**SA**	**MA**	**MD**	**SD**	**Z**	**p**

16. Psychiatry has a low prestige among the general public.	7.9	40.5	41.8	9.8	2.6	53.6	34	9.8	1.36	0.42,ns
17. Psychiatry has a high status among other medical disciplines.	3.3	23.5	64.7	8.5	2	41.8	51.6	4.6	2.64	.008
18. Many people who could not obtain a residency position in other specialties eventually enter psychiatry.	2	16.3	37.3	44.4	0.7	18.3	37.2	43.8	.02	.97,ns
19. Psychiatry is a discipline filled with international medical graduates whose skills are of low quality.	3.9	7.2	45.8	43.1	2	0.7	31.3	66	3.75	.0001
20. My family discouraged me from entering psychiatry.	11.8	19	17.6	51.6	4.6	26.1	26.1	43.2	.18	.85
21. Friends and fellow students discouraged me from entering psychiatry.	9.2	19.6	31.3	39.9	3.3	24.2	30.7	41.8	.84	.39
22. If a student expresses interest in psychiatry, he or she risks being associated with a group of other would-be psychiatrists who are often seen by others as odd, peculiar, or neurotic.	6.5	28.1	30.7	34.7	3.9	24.2	36.6	35.3	.92	.35,ns
23. I feel uncomfortable with mentally ill patients.	8.5	43.8	29.4	18.3	4.6	25.5	35.3	34.6	3.76	.0001

SA = Strongly agree; MA = Moderately agree; MD = Moderately disagree; SD = Strongly disagree.

**Table 6:** Specific medical school factors (percentage of students)

	Before (%)				After (%)				Statistics	
**Question**	**SA**	**MA**	**MD**	**SD**	**SA**	**MA**	**MD**	**SD**	**Z**	**p**

24. Teaching of psychiatry at my medical school is interesting and of good quality.	17.7	54.2	15.7	12.4	46.4	45.1	7.2	1.3	5.64	.0000
25. During my psychiatry rotation, psychiatry residents were good role models.										
26. Attending psychiatrists during my psychiatry rotation were good role models.										
27. Most psychiatrists at my medical school are clear, logical thinkers.	21.6	52.3	26.1	0.0	48.4	47	3.9	0.7	5.24	.0000
28. Most nonpsychiatry and house staff at my medical school are respectful of psychiatry.	10.5	47.7	26.8	15	22.9	56.8	18.3	2	4.53	.0000
29. Although I am interested in psychiatry, no effort was made to encourage my becoming a psychiatrist at my medical school.	24.2	32	23.5	20.3	4.6	23.5	39.9	32	4.82	.0000

SA = Strongly agree; MA = Moderately agree; MD = Moderately disagree; SD = Strongly disagree.

### Overall merits of psychiatry

before the clerkship, most students expressed a positive view about the impact of research in improving care for major psychiatric disorders (93.5%), recognizing psychiatry as a *scientific *(72.5%) and *rapidly expanding frontier of medicine *(76.5%). After the training, students were even more likely to disagree that *psychiatry is unscientific and imprecise *(from 27.5% to 11.7%, p < .001; see Table 1).

### Efficacy

after the clerkship, students were more prone to agree that psychiatric consultation is often helpful either to medical or surgical patients (93.4 to 96%, p - .03), as well as to most people who receive psychiatric treatment (88.8% to 95.4%, p < .0001; see Table 2).

### Role definition and functioning of psychiatrists

Most students believed, with no significant change between assessments, that psychiatrists have more authority and influence in mental health teams, and that psychiatry is a genuine and valid branch of medicine, even if psychiatrist are not too ''biologically'' minded.

After the clerkship, they were more likely to agree that psychiatrists are logical thinkers (71.9% to 85.6%, p = .02), are more qualified than psychologists and social workers (90.8% to 94.8%, p < .01), and are not strongly committed with a too theoretical and psychodynamic approach (90.2% to 97.4%, p < .001).

On the other side, despite a highly significant decrease, half of the students still think that psychiatrists *are too frequently apologetic when teaching *(79.7% to 50%, p < .001; see Table 3).

### Possible abuse and social criticism

after the clerkship, significantly less students agreed that *"psychiatrists abuse their legal power to hospitalize patients against their will*" (from 8.5% to 2.7%, p = .02); there was not a significant change about the opinion that psychiatrists earn less money comparing to doctors from other areas (from 34% to 27.4%; see Table 4).

### Career and personal reward

more than half of students think that psychiatry has a low prestige either among the general public and other medical specialities. In both assessments, around one third of the students reported they were discouraged to pursue psychiatry by their families or fellow students, and that colleagues who are interested in psychiatry are seen as odd, strange, or neurotic. On the other side, students were less likely to agree that *psychiatry is a discipline filled with international medical graduates whose skills are of low quality *(88.9%, p < .001), and reported that they felt much more comfortable with patients after the clerkship (47.7% to 69.9%, p < .001; see Table 5).

### Specific medical school factors

There was a highly significant change in every item of this dimension, suggesting a positive experience with the clerkship. Students were quite satisfied with the quality of teaching (71.9% to 91.5%, p < .0001) and with the encouragement to follow a career in psychiatry (43.8% to 71.9%, p < .00001). They also felt that psychiatrists are logical thinkers (73.9% to 95.4%, p < .0001), and respected by the faculty of the medical school (58.2% to 79.7%, p < .0001; see Table 6).

The intention of students to pursue a career in psychiatry is exposed in Table 7. In both assessments, more than 80% of the students refused or almost refused to consider psychiatry as a future specialty. After the clerkship, there was a significant increase of students decided to take a residency in psychiatry, as well as students considering the possibility to choose psychiatry, although still undecided (Chi-square - 9.62, p = 0.04).

**Table 7:** Medical student's responses about considering a future career in psychiatry

	Before clerkship		After clerkship	
**How seriously are you considering psychiatry as a career choice?**	**n**	**%**	**n**	**%**

I never considered a career in psychiatry	79	51.6	75	49
I considered psychiatry, but I will definitely not enter it	26	17	23	15
I have considered psychiatry, but I will probably not enter it	35	22.9	28	18.3
I am considering psychiatry now, but I am not sure if I will enter it	8	5.2	16	10.5
I will definitely take a residency in psychiatry	5	3.3	11	7.2

## Discussion

To our knowledge, this is the first study addressing the impact of a clerkship in the students' attitude towards psychiatry in Portugal. The study sample is representative of the student population, as it included all subjects approached and there were no dropouts to report. Because data was collected in just one school, results may not be directly applied to other Portuguese medical schools, which is a limitation of the study. The unequal number of male and female students found (39.2% and 60.8%, respectively) is a constant across all grades in our medical school, and reflects the significant predominance of female students currently attending most university degrees in Portugal, namely in medicine.

The results obtained in this study indicate clearly that the clerkship had a favorable overall impact on the student attitude towards psychiatry. Overall, students were more likely to consider psychiatry as a scientific and expanding area of medicine, with a robust level of effectiveness regarding psychiatric treatments. This is a relevant improvement, since the perceived lack of a scientific basis in the psychiatric practice seems to have a harmful influence on the likelihood of choosing psychiatry as a future career [22]. The students' opinion about psychiatrists improved significantly. The students have come to better understand that there is no abuse of legal power in events of compulsory admission, which illustrates how direct contact with psychiatric practice might be effective in shifting previous negative attitudes.

The clerkship experience in the medical school was very rewarding. Students were more likely to report general satisfaction with the teaching quality and with the encouragement to pursue psychiatry, also feeling that psychiatry was respected among the other faculty members. This is a valuable result, as research shows that medical schools specifically may influence recruitment, with the strongest academic centers having the best recruitment rates[23,24]. The fact that they felt much more comfortable with patients after training is also suggestive of a favorable impact of the clerkship on previous stigma concerning psychiatric patients, reinforcing the need for an earlier undergraduate exposure to psychiatry, as pointed out by Eagles [25].

Generally speaking, these results are in agreement with the above mentioned studies (see *Background*) in which an improvement of attitudes among the students was found after the clerkship. When compared with studies using the same methodology, Portuguese students have an overall attitude similar to that of US students [20], and more positive than that of students from Ghana [5] and Spain [21]. Though these differences may be the result of different training models, other factors may not be ignored, namely those resulting from the prevailing culture, the structure of the National Health System and the visibility of psychiatry within that system.

On the other hand, the clerkship had no significant impact in some aspects, in which there was no attitude improvement. This can't be seen as unexpected as it had been observed in several studies with similar results [10,26-28]. After the clerkship, almost half of the students continued to consider that psychiatry has a low prestige, and one third of them also felt some kind of negative pressure from peers and family on the possibility of choosing psychiatry as a future career. Furthermore, almost 30% continued to agree that "if a student expresses interest in psychiatry, he or she risks being associated with a group of other would-be psychiatrists who are often seen by others as odd, peculiar, or neurotic", suggesting that a 'stigmatized' perspective of psychiatry still exists in a significant group of students, and, more importantly, remains after training.

This is in agreement with other investigators who found similar results in Ghana[5] and in Australia[29], thus suggesting the existence of a set of beliefs, regardless of country or socio-economic background. Moreover, these beliefs may be particularly strong within the medical and academic fields: in a survey conducted in Scotland, the low status of psychiatry among other medical doctors was considered to be the single determinant which affected recruitment most negatively[30].

Given these findings, what about the intention to choose psychiatry as a future specialty? Taken as a whole, before the clerkship, 51.6% of the Portuguese students did not consider psychiatry as a future career choice, and a further 17% were determined not to enter the residency. This result is coherent with the frequently reported low popularity of psychiatry as a first-choice career in different settings[3]. The numbers in our sample were even worse than those found in countries where the recruitment of psychiatrists faced a difficult situation in the last years, in which the total number of students rejecting the idea to pursue psychiatry ranged from 38.2% [20] to 47.2% [5]. After the clerkship, it is apparent that although the proportion of 'refusers' remains high, there was a significant increase in both students decided to opt for psychiatry (from 3.3% to 7.2%) and students pondering this possibility but still 'undecided' (5.2% to 10.5%).

Despite these apparently favorable results, we know that the students' attitudes may not be a reliable factor in predicting the future choice of psychiatry as a career. Although some authors claim that the clerkship is the strongest predictor of specialization in psychiatry[31,32], this suggestion has yet to be established[33]. Regarding this issue, Balon suggests that "most of the studies mix up the impact of the clerkship on attitudes toward psychiatry and psychiatry as a career choice", suggesting the need to separate these two aspects and to carry out research based on intervention studies rather than observational studies[34].

Nevertheless, given that specialty preferences before entering a medical school do not appear to deeply influence a definitive career selection [35,36], it could be important to offer student a well-structured and high quality clerkship that may promote early and direct contact with patients in acute and non-acute settings, supervised by mentors which provide good role-models. Furthermore, in countries like Portugal, where there is an intense competition in medical recruitment, the clerkship may be a valuable opportunity to identify and encourage talented students to pursue psychiatry as a realistic choice.

## Conclusions

What are the implications of our results beyond Portugal? Firstly, we have to be cautious with the interpretation and possible inference of the results, as the likely mechanisms by which the clerkship influences the students' attitudes are not completely understood[34]. The existing literature suggests various factors such as the length of the clerkship, quality of training, teaching strategies, perceived quality of patient care, demographic characteristics of medical students, although none appears to be strong enough to stand out from the rest [15,27].

Moreover, in our specific case, results may also have been paradoxically improved by the shortage of previous contact with psychiatry during the medical course. Students only get in touch with psychiatry in the fifth year of medical school, during a series of nine seminars in which direct contact with real psychiatric patients is limited in time. Given the direct experience of the clerkship, allowing for a much deeper contact with patients and professionals, we cannot categorically reject the possibility of some degree of halo-effect among the students, thus introducing a positive bias in the results.

Taking into account these limitations, this study reiterates the findings of studies published in several countries suggesting that the clerkship has and overall positive effect on attitude towards psychiatry. However, this study also strengthens the notion that there are aspects which do not vary significantly with the clerkship, particularly regarding the perception of the prestige of the specialty, as well as the negative pressure to follow psychiatry as a future career option. This finding is of particular importance, as it identifies areas that require a more profound approach in order to improve attitudes and reduce stigmatization.

Lastly, though this is common to other studies in this field yet largely unreported, the clerkship appears not to induce students to a negative perception of psychiatry. Though no precise explanation was found for this, it should nevertheless be highlighted. One possibility is the stark contrast between the negative expectations prior to the clerkship and the rewarding effect of contact with patients and professionals during the clerkship. No studies have been found to address this issue.

Further research should address not only this topic, but also i. the inner mechanisms by which the training experience may induce changes in attitudes towards psychiatry, ii. the factors that preclude changes in some negative believes regarding psychiatry and among future medical doctors, regardless of country and socio-cultural background, and iii. the ethical issues regarding the inadequate use of the clerkship as a kind of "recruitment" device, in which the quality of the clerkship seems more orientated to foster recruitment than to promote better teaching and learning.

Without a more thorough study of these issues, it will prove difficult to find a solution for the most important factor in the issue of recruitment into psychiatry: Should the improvement of attitudes toward psychiatry after training be considered a mere outcome indicator of the clerkship's quality or can it be a reliable indicator of a future option for psychiatry?

## Competing interests

The authors declare that they have no competing interests.

## Authors' contributions

MX prepared the study, administered the surveys, and wrote the first draft of the manuscript. Both authors have reviewed and approved the text of the manuscript.

## Pre-publication history

The pre-publication history for this paper can be accessed here:

http://www.biomedcentral.com/1472-6920/10/56/prepub
